# The closed-mindedness that wasn’t: need for structure and expectancy-inconsistent information

**DOI:** 10.3389/fpsyg.2015.00896

**Published:** 2015-07-02

**Authors:** Markus Kemmelmeier

**Affiliations:** Interdisciplinary Ph.D. Program in Social Psychology, University of Nevada, RenoNV, USA

**Keywords:** person memory, categorical processing, need for structure, intolerance of ambiguity, need for closure, expectancies, social-cognition

## Abstract

Social-cognitive researchers have typically assumed that individuals high in need for structure or need for closure tend to be closed-minded: they are motivated to resist or ignore information that is inconsistent with existing beliefs but instead they rely on category-based expectancies. The present paper argues that this conclusion is not necessarily warranted because previous studies did not allow individual differences in categorical processing to emerge and did not consider different distributions of category-relevant information. Using a person memory paradigm, Experiments 1 and 2 shows that, when categorical processing is optional, high need-for-structure individuals are especially likely to use this type processing to reduce uncertainty, which results in superior recall for expectancy-inconsistent information. Experiment 2 demonstrates that such information is also more likely to be used in judgment making, leading to judgmental moderation among high need-for-structure individuals. Experiments 3 and 4 used a person memory paradigm which requires categorical processing regardless of levels of need for structure. Experiments 3 and 4 demonstrate that, whether expectancy-consistent or -inconsistent information is recalled better is a function of whether the majority of available information is compatible or incompatible with an initial category-based expectancy. Experiment 4 confirmed that the extent to which high need-for-structure individuals attend to different types of information varies with their distribution. The discussion highlights that task affordances have a critical influence on the consequences of categorical processing for memory and social judgment. Thus, high need for structure does not necessarily equate closed-mindedness.

## Introduction

Much research in psychology and related social sciences assumes that people experience ambiguity and uncertainty as aversive and seek to reduce it (e.g., Gudykunst, 1985; Levine, 1985; Hogg, 2000; Inglis, 2000; Kruglanski, 2004). Yet, there are also stark differences between individuals, with some being much more tolerant of and others much more motivated to reduce any uncertainty (e.g., Mclain, 1993; Webster and Kruglanski, 1994; Sorrentino and Roney, 2000; Thompson et al., 2001). Generally, theorists suggest that flight from ambiguity and uncertainty implies closed-mindedness, which can be defined as obstinate resistance to unfamiliar or unwelcome ideas (e.g., Merriam-Webster’s online dictionary, n.d.). Closed-minded individuals cling to existing values and beliefs whereas new experiences and new information, especially when they challenge existing ideas, are discounted, if not altogether ignored (Driscoll et al., 1991; Dijksterhuis et al., 1996; Kruglanski, 2004). The present paper challenges the idea that seeking to replace uncertainty with cognitive closure and structure necessarily implies closed-mindedness. The same characteristics that may make individuals affirm existing beliefs, namely an overreliance on categorical processing of social information, might also make them more attentive to new and unexpected information. The goal of the present research is to show under which circumstances individual differences in dispositional intolerance for ambiguity and need for structure make individuals more or less open to unexpected information.

Early research on individual differences in closed-mindedness focused on intolerance of ambiguity and pointed to this concepts’ association with phenomena such as authoritarianism, ethnocentrism and stereotyping (e.g., Rokeach, 1948; Frenkel-Brunswik, 1949; Block and Block, 1951). Though modern research has supported some of its conclusions (e.g., Kemmelmeier, 2007, 2010), this work was criticized based on both methodological and theoretical grounds (e.g., Furnham and Ribchester, 1995). Yet, to date there are two well-established lines of research in social and personality psychology that carry forth this tradition.

First, research on need for structure has demonstrated that people vary in their inclination to reduce complexity when thinking about and interacting with their social environment (Neuberg and Newsom, 1993; Thompson et al., 2001). Because they abhor ambiguity and lack of structure, those individuals high in need for structure are more inclined to rely on stereotypes (Neuberg and Newsom, 1993; Newheiser and Dovidio, 2012), and are also more likely to acquire new stereotypes (Schaller et al., 1995), even when presented with unbiased information (Gordon, 1997). Reliance on stereotypes and categorical representations of others simplifies and structures social-cognition, reducing the processing effort that is required (e.g., Brewer, 1988; Fiske and Neuberg, 1990; Fiske et al., 1999). Further, this reduces uncertainty by activating stereotypic knowledge, which allows individuals to anticipate the behaviors and features of others (Macrae and Bodenhausen, 2000). Consistent with the idea that those motivated by a high need for structure rely heavily on social categorization, Moskowitz (1993) demonstrated that such individuals are more likely to make trait inferences based on behaviors than individuals who are low in need for closure. In other words, high need-for-closure individuals readily conclude that someone who, for instance, won a chess tournament and graduated magna cum laude must be member of the category of “intelligent people” (see also Webster and Kruglanski, 1994).

The second, related line of research concerns the construct of need for closure, that is, epistemic motivation to have any answer on a given topic rather than expose the individual to continued ambiguity (Kruglanski and Webster, 1996; Kruglanski, 2004). This research assumes that all individuals are motivated to reduce uncertainty; yet, people who are high in a dispositional need for closure tend to reduce the feelings of discomfort induced by uncertainty by relying on category-based processing (e.g., Brewer, 1988; Fiske et al., 1999). By contrast, individuals low in need for closure are assumed to reduce uncertainty by engaging in detailed, effortful processing, which avoids the generalizations that result from viewing others as exchangeable members of a social category. Following vigorous debate (Kruglanski et al., 1997; Neuberg et al., 1997a,b, contemporary research now treats the need for structure construct and the need for closure construct as largely exchangeable (e.g., Jost et al., 1999; Bouckenooghe et al., 2007).

Dijksterhuis et al. (1996) have thus far produced the most compelling evidence that need for closure implies closed-mindedness, here conceived as individuals turning away or even ignoring new and unexpected information. The authors provided participants with general descriptions about a target group (e.g., hooligans). Participants then read descriptions of 15 members of this group, five of which were engaged in expected behavior (e.g., starting a bar fight), five of which were engaged in unexpected behavior (e.g., doesn’t drink alcohol) and five of which were unrelated. Across two studies, Dijksterhuis et al. (1996) documented that participants low in need for closure were more attentive to and had a better memory for behavior that was inconsistent with their initial expectancy. This replicated a well-established finding based on which expectancy-inconsistent information triggers effortful cognitive processing which, in turn, makes it more memorable and more likely to be used in subsequent judgments (Hastie and Kumar, 1979; Stangor and McMillan, 1992). However, participants high in need for closure focused more on and were more likely to recall behavior that confirmed their initial expectancy, but ignored evidence that contradicted their initial beliefs about the group (see Driscoll et al., 1991 for similar findings involving differences in uncertainty orientation). Because expectancy-inconsistent information loomed larger for low need-for-closure participants, they evaluated the group as less stereotypical than high need-for-closure individuals.

The research reported here reexamines the generality of the findings of Dijksterhuis et al. (1996). The focus is on need for structure, which is measured using the 11-items Personal Need for Structure scale (PNS; Neuberg and Newsom, 1993). The PNS scale is composed of two separate factors: *Desire for Structure* (DFS) reflects participants’ dispositional motivation to create structure in their lives, whereas *Response to Lack of Structure* (RLS) taps responses to unstructured, unpredictable and generally ambiguous situations. Though much research combines both factors into one, studies have documented distinct correlates of the two subscales (e.g., Cavazos et al., 2012).

In the present research, the focal interest is on DFS. To the extent that individuals encounter information about a target that is ambiguous (e.g., because different pieces of information lead to contradictory conclusions), those high in DFS should be highly motivated to restructure the information, that is, to arrive at a simple cognitive structure. Arguably, when forming an impression about a target person this means categorizing him based on the available information (Moskowitz, 1993). If a critical mass of the information suggests that the target is intelligent (or not intelligent), this allows perceivers to bring order into their experiences. By focusing only on part of what is known about the person, a perceiver who seizes on information suggesting that the person is intelligent has a chance to categorize him as “a smart person,” and thus simplify their representation of the person. Given their inclination to reduce complexity in thinking about others, high-DFS participants should be more inclined than low-DFS individuals to categorize such a newly encountered target.

To the extent that high-DFS participants readily categorize a target person, this should bring to mind characteristics that are typical for a member of this category. This category-based information defines participants’ expectancies as to what other information they might learn about the target person (e.g., Olson et al., 1996). For instance, participants might expect “a smart person” to have a stellar educational record. If they learn that the person dropped out of high school, there is a need for them to respond to this unexpected information. Researchers have long argued that such information prompts efforts to reconcile it with one’s expectancy, with the resulting cognitive elaboration making expectancy-inconsistent information more memorable than expectancy-consistent information (e.g., Hastie and Kumar, 1979; Stangor and McMillan, 1992; but see Heider et al., 2007). As a consequence, because they are more likely to engage in categorical processing of a target, high-DFS should be more attentive to expectancy-inconsistent information than low-DFS individuals, and remember it better.

Recall that Dijksterhuis et al. (1996) did not obtain the pattern of findings anticipated here. These authors found that high need for structure *reduced* the attention paid to expectancy-inconsistent information. One possible reason is that Dijksterhuis et al. (1996), like many others (e.g., Srull et al., 1985; Driscoll et al., 1991), provided participants with advance information about the target, which primed a social category, and thus established category-based expectancies *prior* to participants encountering any additional information. That is, Dijksterhuis et al. (1996) prompted categorical processing on the part of all participants regardless of their level of need for structure. As a result, these authors were able to examine whether high and low need for structure participants would process expectancy-consistent and expectancy-inconsistent information differently once categorical processing was engaged, but not if participants would engage in categorical processing or not.

The paradigm employed in the Experiments 1 and 2 differs from Dijksterhuis et al. (1996), though it uses another common approach in research on impression formation (e.g., Hastie and Kumar, 1979; Hemsley and Marmurek, 1982; Bargh and Thein, 1985). Rather than providing participants with category labels in advance, expectancies are induced indirectly through the distribution of the information made available to participants. If the majority of the available information implies that the target is intelligent, but only a minority of information implies he is not intelligent, then participants arrive at the overall impression that the target is intelligent, i.e., they may categorize the target as “a smart person.” This approach is especially likely to render information memorable that is *in*consistent with the overall impression conveyed by the majority of information (Stangor and McMillan, 1992). Research does not always differentiate whether participants are provided with an explicit categorization or whether a suitable category has to be inferred by participants (but see Neuberg et al., 1997b). However, for Experiments 1 and 2 the distinction is crucial: if high-DFS individuals are more inclined to engage in categorical processing than their low-DFS counterparts, this should only become apparent when categorical processing is optional and the task does not constrain whether effortful (piecemeal) processing or categorical processing is deployed. As demonstrated later in Experiments 3 and 4, requiring both high-DFS and low-DFS participants to engage in categorical processing may engage different processes and result in different effects on memory.

Another contrast between the present research and Dijksterhuis et al. (1996) is that these authors focused on groups, whereas the present focus is on individuals. All members of a group are not necessarily expected to behave in the same way. Yet, people do expect a person to behave consistently; that is, a person who is smart should also be able to finish high school. Arguably, a person about whom expectancy-inconsistent information becomes known seems to prompt much more intense cognitive efforts to reconcile new information with existing one than is the case of groups, rendering inconsistent information about individuals more memorable (e.g., Stern et al., 1984; Srull et al., 1985).

## Experiment 1

In the first experiment participants were provided with a majority of information that was intended to induce the overall impression that the target person is either intelligent or unintelligent. A subsequent recall task and a recognition task were expected to reveal that participants high in DFS would more likely to engage in categorical processing, resulting in better memory for expectancy-inconsistent information. Thus, the prediction was:

Hypothesis 1: Compared to low-DFS individuals, high-DFS individuals are more attentive to expectancy-inconsistent information than expectancy-consistent information, and remember it better.

It was also anticipated that those high in DFS would exhibit evidence of categorical processing on the recognition task. This should prompt high-DFS participants to falsely accept information as previously seen if this information that was directly relevant to the overall impression. That is, they would produce more false alarms concerning such information, but not neutral information.

Hypothesis 2: Compared to low-DFS individuals, high-DFS individuals are more likely to exhibit in response biases on a recognition memory task in favor of expectancy-relevant information relative to neutral behaviors.

### Participants

One hundred twenty-one undergraduates participated in this research in exchange for partial course credit (52% women, average age 18.7 years, range 17–23). At the beginning of the term (2–6 weeks prior to the experiment), all students enrolled in a large introductory psychology course (*n* > 1,100) completed a six-item version (items 3, 4, 6, 8, 9, and 11; α = 0.86) of the PNS scale (Neuberg and Newsom, 1993), which correlated well with the original 11-items scale (*r* = 0.88). Participants were recruited based on whether their scores placed in them in the top (*n* = 62) or bottom (*n* = 59) 20% of the distribution^1^.

### Materials

Based on Fuhrman et al. (1989), 24 behavior descriptions implying high intelligence were selected (average intelligence ratings 7.4–8.9 on a nine-point Likert type scale). Sample items included “Was voted most likely to succeed by members of his class” and “Won a chess tournament.” Similarly, we selected 24 behavior descriptions implying lack of intelligence (average intelligence ratings 2.2–4.4 on the same scale). Sample items included “Kept the windows open while running the air conditioner” and “Flunked an aptitude test.” Also selected were 12 neutral items with middling intelligence ratings (average rating 4.9–5.9). A comparison of normativity ratings, also by Fuhrman et al. (1989), showed no difference between the three types of behaviors.

Two different stimulus sets were constructed based on half of all behaviors selected from Fuhrman et al. (1989). The first stimulus set intended to convey the impression that Bob was intelligent and included 12 intelligent behaviors, six unintelligent behaviors and six neutral behaviors, all randomly chosen from their respective pools (high intelligence list). A second stimulus set conveyed that Bob was unintelligent and included 12 unintelligent, six intelligent and the same six neutral behaviors, chosen from their respective pools of items (low intelligence list). The smaller set of six behaviors of one list always was a subset of the larger set of 12 behaviors of the other list. There were two variants of the same stimulus set to ensure that, across participants, each behavior was presented equally often as part of the set of six and as part of the set of 12 behaviors. [The other half of the behaviors selected from Fuhrman et al. (1989) was used to generate foils for the recognition task.]

### Procedure

Upon arrival to the laboratory, participants were told that they would be participating in a study, in which they were to form an impression about a target person (“Bob”). They then saw 24 behavior descriptions pertaining to that person. Half of the participants saw the behaviors of the high intelligence list, and half the behaviors of the low intelligence list. The majority of behaviors (here: half of all behaviors presented) was expected to determine the overall impression of Bob as intelligent or not intelligent (see also Hastie and Kumar, 1979). For half of the participants these 24 behaviors were provided simultaneously in random order on a computer screen, with participants having as much time as they wished to study the list. For the other half, the 24 behaviors were presented sequentially on a computer screen, such that the each behavior appeared for 5 s, followed by a 1-s blank screen. Within each presentation condition, stimulus order was counterbalanced, with half seeing the reverse order of the other half.

Following the presentation of behavior descriptions, and after a 2-min distractor task, participants were given 3 min to recall as many of Bob’s behaviors as they could. Immediately afterward, participants were asked to work on a recognition task in which they were presented with the 24 previously viewed behaviors intermingled with 24 new behaviors. That is, beyond the 12 old impression-defining behaviors participants saw 12 new, previously unseen ones; similarly, the six impression-inconsistent behaviors were supplemented by six new behaviors of the same kind, and the six previously viewed neutral behaviors were presented alongside six new ones. All 48 behaviors were presented in random order on a computer screen, and participants indicated through predefined key entries whether they had seen the behavior before or not. However, because of equipment malfunction or researcher error, 16 of 121 participants did not complete this task.

Once the recognition task was completed, participants completed the 11-items PNS scale (α = 0.90), based on which the four-item DFS subscale (α = 0.88) and the seven-item RLS subscale (α = 0.84) were computed (Neuberg and Newsom, 1993). Participants also completed the 22-item McLain tolerance of ambiguity scale (α = 0.85), which did not qualify results, and is thus not reported further. Finally, participants were thanked, debriefed and dismissed.

### Results

#### Recall

Participants recalled between 4 and 14 behaviors (*M* = 9.60). Recall protocols were subsequently coded by two coders using the gist criterion; that is, they were coded if the general content and the valence of the behavior was preserved (e.g., Hastie and Kumar, 1979; Srull et al., 1985; Driscoll et al., 1991). Disagreements between coders were resolved through discussion. Recalled behaviors were categorized by whether they were part of the 12 behaviors defining the overall impression, whether they were inconsistent with it, or whether they were neutral. The number of recalled behaviors within each category was subsequently divided by the number of behaviors presented, and the resulting proportions submitted to a 2 (PNS: high vs. low) × 2 (Majority of Behaviors: Intelligent vs. unintelligent) × 2 (Presentation: simultaneous vs. sequential) × 2 (Order) × 3 (Behavior Type: impression-defining, inconsistent, neutral) mixed factorial model with the last factor varying within participants.

**FIGURE 1:**
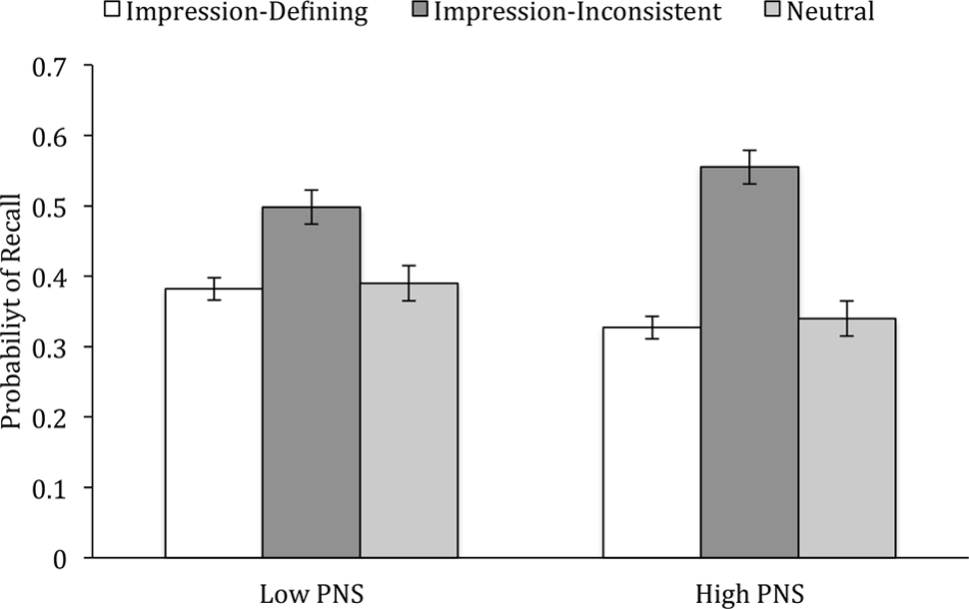
**Likelihood of recall of impression-defining, impression-inconsistent, and neutral behaviors as a function of PNS (Experiment 1).** Bars reflect one standard error above and below the mean.

A main effect for Behavior Type, *F*(2,104) = 37.39, *p* < 0.001, $\eta_{p}^{2}$ = 0.42, showed that impression-inconsistent behaviors were more likely to be recalled (*M* = 0.53) than either impression-defining (i.e., majority) behaviors (*M* = 0.37) or neutral behaviors (*M* = 0.37). Critically, this effect was qualified by a PNS x Behavior Type interaction, *F*(2,104) = 3.96, *p* = 0.022, $\eta_{p}^{2}$ = 0.07. As displayed in **Figure 1**, high-PNS individuals were surprisingly less likely to recall impression-defining behaviors, *p* = 0.016, but, as predicted by Hypothesis 1, somewhat more likely to recall impression-inconsistent behaviors than low-PNS individuals, *p* = 0.098, though there was no difference in the recall of neutral behaviors, *p* = 0.16. The only other effect emerged for Order, *F*(1,105) = 4.10, *p* = 0.046, $\eta_{p}^{2}$ = 0.04, which did not interact with any other experimental factor, and was thus inconsequential.

This analysis was repeated by replacing the dichotomous PNS variable with alternating the DFS scale or RLS subscales as continuous predictor within the same general model. Though both subscales were substantially correlated (*r* = 0.72), the above two-way interaction with Behavior Type only emerged for DFS, *F*(2,104) = 3.25, *p* = 0.043, $\eta_{p}^{2}$ = 0.06, but not RLS, *F*(2,104) = 0.65, *p* = 0.53, $\eta_{p}^{2}$ = 0.01. (Outside of the previously reported Order effect, no other effects were significant.) Consistent with Hypothesis 1, this pattern suggests that the differential recall of impression-defining and -inconsistent items was mainly a function of individual differences in DFS.

#### Recognition

A first inspection revealed that correct identification of previously seen behaviors was high (95% hits on average) across all behavior types, with false identifications of previously unseen behaviors being low (on average 2% false alarms). In the signal detection analysis, *A’* served as non-parametric measure of sensitivity, with 1 reflecting that participants distinguished perfectly between previously seen and unseen items, and 0.50 indicating the lack of any distinction between the two kinds of items (Stanislaw and Todorov, 1999). *B”* served as measure of response bias ranging from -1 (extreme bias in favor of “previously seen” responses) to +1 (extreme bias in favor of “not previously seen” responses), and 0 reflecting the absence of any bias.

##### Sensitivity (A’)

Using the above factorial model, a main effect for Behavior Type, *F*(2,86) = 4.36, *p* = 0.016, $\eta_{p}^{2}$ = 0.09, indicated that sensitivity was at least somewhat greater for both impression-defining behaviors (*M* = 0.983) and impression-inconsistent behaviors (*M* = 0.987) compared to neutral behaviors (*M* = 0.977), *p* = 0.08 and *p* = 0.004, respectively^2^. A Behavior Type x Majority of Behaviors interaction, *F*(2,86) = 5.11, *p* = 0.008, $\eta_{p}^{2}$ = 0.11, showed that this pattern primarily occurred when the target person was presented as being of high intelligence. A PNS by Majority of Behaviors interaction, *F*(1,87) = 6.04, *p* = 0.016, $\eta_{p}^{2}$ = 0.06, revealed that high-PNS participants were less sensitive to the presence of previously seen vs. unseen items when the majority of behaviors implied that Bob was intelligent rather than unintelligent. However, this was the case irrespective of Behavior Type, with all other effects involving PNS being non-significant, all *F* < 2.82, *p* > 0.096, $\eta_{p}^{2}$ < 0.04. Similar findings were obtained when the dichotomous PNS variable was replaced by either the continuous DFS or RLS predictors, though RLS never interacted with Majority of Behavior. Altogether, DFS was not related to any differential sensitivity of recognition memory for impression-consistent and impression-inconsistent information.

##### Response bias (B”)

A Behavior Type main effect, *F*(2,86) = 3.70, *p* = 0.029, $\eta_{p}^{2}$ = 0.08, showed that for impression-consistent behaviors (*M* = 0.25) and neutral behaviors (*M* = 0.29) there was less of a tendency to classify an item as not previously seen than for impression-inconsistent items (*M* = 0.11). Recall that high PNS participants were expected to recognize even previously unseen behaviors as seen if they were impression-relevant, simply because they would be more likely to rely on categorical processing; that is, for this group *B”* was expected to be *lower* for impression relevant behaviors. Though the Behavior Type × PNS interaction failed to reach significance, *F*(2,86) = 1.73, *p* = 0.18, $\eta_{p}^{2}$ = 0.04, the three-way interaction also involving Majority of Behavior was reliable, *F*(2,86) = 4.87, *p* = 0.010, $\eta_{p}^{2}$ = 0.10. As was confirmed in a subsequent contrast analysis (*p* = 0.027), for high-PNS participants exposed a majority of unintelligent behaviors, *B”* was lower for impression-relevant behaviors (*M*_consistent_ = 0.21, *M*_inconsistent_ = -0.08) than neutral behaviors (*M* = 0.36); yet, this pattern did not emerge for any other combination for PNS and Majority of Behavior. Aside from the otherwise inconsequential observation that simultaneous presentation of the behaviors resulted in a higher *B”* than sequential presentation, *F*(1,87) = 4.18, *p* = 0.044, $\eta_{p}^{2}$ = 0.05, no other effects were significant.

When PNS status was replaced with DFS, aside from the Behavior Type main effect, *F*(2,86) = 3.64, *p* = 0.030, $\eta_{p}^{2}$ = 0.08, a trend-level Behavior Type x DFS interaction, *F*(2,86) = 2.61, *p* = 0.079, $\eta_{p}^{2}$ = 0.06, confirmed that for high DFS participants (+1 SD above mean) *B”* for impression consistent (*M* = 0.11) and impression-inconsistent behaviors (*M* = 0.05) was higher than for neutral items (*M* = 0.33), *p* = 0.045 and *p* = 0.006, respectively. No such pairwise differences emerged for low DFS participants (-1 SD below mean; *M* = 0.36 and 0.15 vs. *M* = 0.22, both *p* > 0.20). As before, a Behavior Type × PNS × Majority interaction, *F*(2,86) = 4.13, *p* = 0.019, $\eta_{p}^{2}$ = 0.09, indicated that this pattern occurred unevenly across the design, and was even further qualified by Presentation Order, *F*(2,86) = 3.91, *p* = 0.024, $\eta_{p}^{2}$ = 0.08. Whereas these findings yield partial support for Hypothesis 2, note that the model in which DFS was replaced with RLS yielded similar findings. The Behavior Type x RLS interaction approached significance, *F*(2,86) = 3.01, *p* = 0.055, $\eta_{p}^{2}$ = 0.07 (being further qualified by Majority of Behavior, *F*(2,86) = 4.41, *p* = 0.015, $\eta_{p}^{2}$ = 0.09), thus confirming the above-described pattern. Overall, analyses of response bias provide at least some corroboration for Hypothesis 2. Participants high in DFS are inclined to engage in categorical processing such that they tend to falsely recognize behaviors that are relevant to their overall impression of the target.

## Experiment 2

The second experiment sought to replicate the recall memory findings of Experiment 1, and to examine whether the greater attentiveness of high-DFS participants would extend to judgments about the target (cf. Dijksterhuis et al., 1996). Judgments are critical in that they often shape one’s behavior toward a target, thus rendering recalled information relevant to social interaction. If high-DFS participants are more likely to engage in categorical processing, they should not only attend to impression-inconsistent information more and recall it better, but rely on it more in their evaluations, and thus produce more moderate judgments.

Hypothesis 3: Compared to low-DFS individuals, to the extent that high-DFS individuals recall impression-inconsistent information better than impression-consistent information, they are less extreme in their judgments.

### Participants

One hundred-two undergraduate students participated in this research in exchange for course credit (31% women, average age 19.0 years, range 17–24).

### Materials and Procedure

Materials were identical to Experiment 1. However, the presentation of behaviors of Bob was simplified with all participants receiving a mostly high intelligence set (1/2 intelligent, 1/4 unintelligent) or a mostly low intelligence set (1/2 unintelligent, 1/4 intelligent) of behaviors as part of a booklet, all in the same order. Participants were asked to study the list until they felt they knew what kind of person Bob is (typically less than 3 min). Prior to a 2-min distractor and the subsequent recall task, participants were asked to rate Bob on seven different trait dimensions using a seven-point Likert-type rating scale. Three traits were used to form an intelligence score [intelligent, bright, simple-minded (reversed), α = 0.66]. [The remaining traits (forgetful, likable, interesting, boring) did not yield any relevant results.] Then, participants worked on the same recall task as in Experiment 1, and completed the PNS scale (α = 0.79), based on which the DFS subscale (α = 0.70) and the RLS subscale (α = 0.71) were computed.

### Results

#### Judgments

Intelligence scores were entered into an analysis with (Majority of Behaviors: Intelligent vs. unintelligent) as between-groups factor and alternatingly the two PNS subscales as continuous predictor. The model involving DFS produced the expected main effect for Majority of Behaviors, such that participants who had learned about more intelligent behaviors than unintelligent behaviors of Bob rated him as more intelligent compared to participants who had learned about more unintelligent than intelligent behaviors (*M* = 5.02 vs. 4.29), *F*(1,98) = 13.82, *p* < 0.001, $\eta_{p}^{2}$ = 0.12. However, a Majority of Behaviors x DFS interaction, *F*(1,98) = 3.82, *p* = 0.053, $\eta_{p}^{2}$ = 0.04, revealed that among individuals low in DFS (1 SD below mean) the effect of Majority of Behaviors was pronounced (*M* = 5.03 vs. 3.95), *p* < 0.001; yet, among those high in DFS (+1 SD above mean), Majority of Behaviors was practically irrelevant for intelligence ratings (*M* = 5.02 vs. 4.69), *p* = 0.22. In other words, individuals high in DFS were less swayed by the preponderance of intelligent or unintelligent behaviors. Parallel analyses for RLS did not replicate this interaction, supporting that effects are unique to DFS.

#### Recall

Protocols were coded and analyzed as in Experiment 1 (average behaviors recalled *M* = 8.58, range 0–15). Proportions of impression-defining, -inconsistent and neutral items were submitted to a principal component analysis, which included Behavior Type and Majority of Behaviors as categorical predictors and again either DFS or RLS as continuous predictors. Across both models (i.e., including DFS or RLS), impression-inconsistent behaviors were more easily recalled (*M* = 0.46) than either impression-defining (*M* = 0.33) or neutral (*M* = 0.31) behaviors, both *F*(2,97) > 23.54, *p* < 0.001, $\eta_{p}^{2}$ > 0.32. The model including DFS replicated the Behavior Type x DFS interaction previously obtained in Experiment 1, *F*(2,97) = 3.59, *p* = 0.031, $\eta_{p}^{2}$ = 0.07. Participants high in DFS (+1 SD above mean) were more likely to recall impression-inconsistent behaviors than those low in DFS (-1 SD below mean; *M* = 0.51 vs. 0.41), *p* = 0.016, though no comparable difference was obtained for impression-defining behaviors (*M* = 0.32 vs. 0.33), *p* = 0.83, and neutral behaviors (*M* = 0.31 vs. 0.32), *p* = 0.83. The equivalent interaction was not significant in the model involving RLS, *F* < 1, $\eta_{p}^{2}$ = 0.02.

#### Relationship between Judgment and Recall

Hypothesis 3 assumes that judgments and recalled behaviors are related, presumably because participants base their ratings on the behaviors that they retained from studying the list (see Stern et al., 1984). To address this possibility, we divided the number of impression-consistent behaviors recalled by the number of impression-inconsistent behaviors recalled. (Here and elsewhere in this report, this ratio could not be computed for participants who did not recall any inconsistent behaviors). The resulting ratio correlated positively with the intelligence score when the majority of behaviors created the impression that Bob is highly intelligent, *r*(47) = 0.34, *p* = 0.020, but negatively when the majority of behaviors created the impression that Bob is unintelligent, *r*(49) = -0.31, *p* = 0.032, suggesting a close link between judgment and recall memory^3^. This pattern is consistent with the idea that, even though the majority of behaviors implied high intelligence, high-DFS individuals rated Bob’s intelligence to be lower because they had a relatively better memory for his unintelligent behaviors than low-DFS individuals. Conversely, when the majority of behaviors implied low intelligence, high-DFS individuals rated Bob’s intelligence to be higher presumably because they retained relatively more information about this intelligent behavior.

## Experiment 3

Experiments 1 and 2 demonstrated that those high in DFS are highly attuned to inconsistent information when they learned about a person’s behaviors. Unlike the studies reported by Dijksterhuis et al. (1996), participants did not have any particular expectancy about the target, which allowed these experiments to reveal whether individuals high or low in DFS would vary in their inclination to engage in social categorization.

But what if individuals already hold an expectancy as they confront situations with different distributions of information? That is, participants may hold category-based expectancies about a target, but the actual characteristics of the target might either confirm or disconfirm the initial expectancy. Experiments 3 and 4 explores the consequences of individual differences in DFS for this type of situation. Specifically, as part of Experiment 3, participants learned that the target person (Bob) is either highly intelligent or clearly below average intelligence. Subsequently, participants were provided with the same 24 behaviors as in Experiment 2, but in one condition the majority of behaviors confirmed the prior expectancy, whereas in another condition the majority of behaviors contradicted the prior expectancy.

Based on the earlier analysis, advance categorization of Bob as smart person or not-so-smart person should now trigger categorical processing on the part of all participants, regardless of whether they are high or low in DFS. Still, a DFS should motivate individuals to hold on to an initial category-based expectancies as much as possible, as not doing so would only open them up to uncertainty and ambiguity (Neuberg and Newsom, 1993; Kruglanski, 2004). Specifically, in extension of Dijksterhuis et al. (1996), one might predict that for individuals high in DFS preexisting expectancies will direct attention to expectancy-consistent information rather than expectancy-inconsistent information, and produce superior memory for the former and inferior memory for the latter type of information. Moreover, high desire-for-structure individuals should now arrive at more stereotypical judgments. In other words, having a category-based expectancy might allow for closed-mindedness to emerge, such that high desire-for-structure individuals are motivated to confirm an initial expectancy in part by ignoring unexpected information (cf. Kruglanski, 2004).

Yet, as amply demonstrated in the literature, the consequences of categorical processing for memory and judgment vary with the circumstances (see Macrae and Bodenhausen, 2000 for a review). For instance, reviews of the last two and a half decades have routinely highlighted that there is considerable situational variability in whether expectancy-consistent and expectancy-inconsistent information is remembered better (e.g., Rojahn and Pettigrew, 1992; Stangor and McMillan, 1992; Fyock and Stangor, 1994; Macrae and Bodenhausen, 2000; Skowronski et al., 2013). Specifically, Sherman et al.’s (1998) “encoding flexibility” model argues that category-based expectancies serve to enhance the efficiency of information processing. Though expectancy-consistent information is typically processed more easily, such information may receive only a minimum of attention, simply because a perceiver does not invest attentional resources unnecessarily in the processing of information whose gist is already known. Rather, attentional resources are devoted to information that is new and unexpected (see also Johnston and Hawley, 1994). As Hilton et al. (1991) put it: “When perceivers receive information that is initially consistent with their expectations, they often assume that their expectations are correct and quickly allocate their attention elsewhere.”

High DFS motivates individuals not only to engage in categorical processing (as demonstrated in Experiments 1 and 2) but also to rely on social categories in their perception of the world. But even though social categories give rise to category-based expectancies, such expectancies may not necessarily direct attention to expectancy-confirmation information. Rather, the consequences of category-based processing should be sensitive to the nature and distribution of the available information. That is, whether high DFS enhances attention to and subsequent memory of expectancy-consistent or expectancy-inconsistent information should depend on whether most of the available information supports or contradicts the expectancies.

Category-based expectancies should render especially individuals with a high DFS particularly sensitive to information that is *in*consistent with their expectancy when the majority of the available evidence supports the expectancy. When individuals expect Bob to be intelligent, but realize that most of Bob’s behaviors support this very idea, they are likely to shift attention away from what they feel they know already to that which is new and surprising. The expected pattern is similar to what was observed in Experiments 1 and 2. Yet, it is clear that any differences between high and low DFS individuals are not due to whether they engage in categorical processing or not since the task requires all participants to do so. Thus, the prediction is:

Hypothesis 4: When the majority of available information confirms an initial category-based expectancy, high-DFS individuals are more attentive to expectancy-*in*consistent information than expectancy-consistent information, and remember it better than is the case for low-DFS individuals.

When, however, cursory processing reveals that the majority of the available information does *not* match individuals’ category-based expectancies, attention should shift to expectancy-consistent information. When participants expect Bob to be intelligent, but face an array of diverse information which does not support this notion, especially those who are high in DFS are likely to attend to information that supports Bob’s intelligence. The prospect of having their existing beliefs overturned should motivate these individuals to hold on to their categorical expectancy, allowing them to avoid ambiguity and uncertainty. Thus, the prediction is:

Hypothesis 5: When the majority of available information does not confirm an initial category-based expectancy, high-DFS individuals are more attentive to expectancy-consistent information than expectancy-*in*consistent information, and remember it better than is the case for low-DFS individuals.

Interestingly, in the Dijksterhuis et al. (1996) paradigm two-thirds of the available information either disconfirmed participants’ category-based expectancy or was neutral, with only a minority (one third of items) being consistent with the expectancy. Hence, similar to what is anticipated in Hypothesis 5, to reduce uncertainty Dijksterhuis et al.’s (1996) high need-for-closure participants might have relied on their expectancy to seek out and dwelled on expectancy-consistent information. This resulted in better memory of and greater weight assigned to expectancy-consistent information.

### Participants

Eighty undergraduates participated in exchange for partial course credit (43% women, average age 19.0 years, range 18–23).

### Materials and Procedure

Materials were identical to the previous experiments. Again, participants were instructed to form an impression about a target person named Bob. However, before participants either received the high intelligence list (12 intelligent, 6 unintelligent, 6 neutral behaviors) or low intelligence list (12 unintelligent, 6 intelligent, 6 neutral behaviors), they received some general information about what kind of person Bob is (adapted from Driscoll et al., 1991). In the high expectancy condition participants read: “In fact, Bob is very intelligent. His sharp quick mind has always helped him to excel at almost anything he does.” The low expectancy condition read: “In fact, Bob is of below average intelligence. His mind works slowly and has always prevented him from excelling at almost anything he does.”

Following the same distractor task as in Experiment 1, participants engaged in the 3-min recall task. On the next page in the booklet participants rated Bob on 11 trait dimensions, which included the seven used in Experiment 2 plus shy, honest, outgoing and smart. Four intelligence-related ratings were combined into an intelligence score [intelligent, bright, smart, simple-minded (reversed); α = 0.76]. (Again, other ratings did not yield pertinent results.) Later in the session, participants completed the PNS scale (α = 0.76), based on which again the DFS (α = 0.70) and the RLS scores (α = 0.66) were generated.

### Results

#### Recall

Protocols were coded and analyzed as before (average behaviors recalled *M* = 7.61, range 1–16). Proportions of recalled behaviors were submitted to a 2 (Expectancy: high vs. low intelligence) × 2 (Majority of Behaviors: intelligent vs. unintelligent) × 3 (Behavior Type: expectancy-consistent, expectancy-inconsistent and neutral) factorial analysis, in which Behavior Type was a repeated-measures factor and alternatingly DFS or RLS were used as continuous predictors.

**FIGURE 2:**
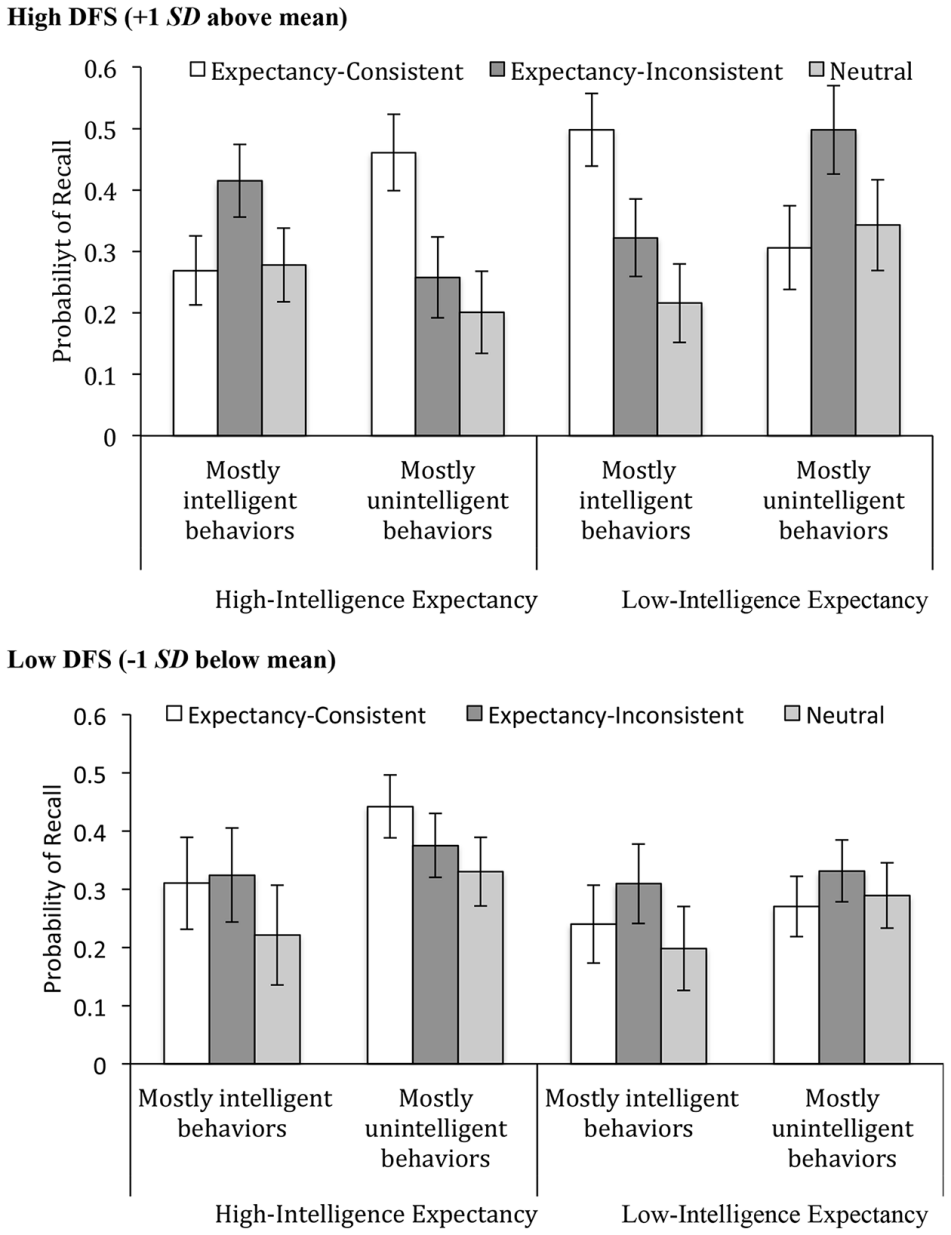
**Likelihood of recall of expectancy-consistent, expectancy-inconsistent, and neutral behaviors as a function of expectancy and majority of behaviors (Experiment 3).** Bars reflect one standard error above and below the mean.

Across both models a main effect of Behavior Type emerged, both *F*(2,71) > 7.51, *p* < 0.002, $\eta_{p}^{2}$ > 0.17, showing that expectancy-consistent and expectancy-inconsistent behaviors were equally likely to be recalled (*M* = 0.35 and 0.35), though more than neutral behaviors (*M* = 0.26). In both models there was also a Behavior Type x Expectancy x Majority of Behaviors three-way interaction, both *F*(2,71) > 5.98, *p* < 0.004, $\eta_{p}^{2}$ > 0.14. However, only in the model including DFS was this three-way term further qualified by a four-way interaction, *F*(2,71) = 3.69, *p* = 0.030, $\eta_{p}^{2}$ = 0.09. The diagnosis of this four-way interaction focuses exclusively on participants high in DFS (+1 SD above mean) because for low-DFS participants (-1 SD below mean), there was no evidence of differential recall of behavior types, all pairwise comparisons *p* > 0.15. As displayed in **Figure 2**, when high-DFS participants expected Bob to be highly intelligent, and the majority of behaviors supported this, high-DFS participants were more likely to recall expectancy-inconsistent (i.e., unintelligent) behaviors than expectancy-consistent or neutral behaviors, *p* = 0.049 and *p* = 0.037, respectively (**Figure 2**, leftmost panel). Likewise, when participants expected Bob to be of low intelligence, and the majority of behaviors supported this idea, high-DFS participants were more likely to recall expectancy-inconsistent (i.e., intelligent) behaviors than either expectancy-consistent or neutral ones, *p* = 0.035 and *p* = 0.054, respectively (**Figure 2**, rightmost panel). Compared to their low-DFS counterparts, high-DFS participants recalled a greater share of expectancy-inconsistent information when given a low-intelligence expectancy (*M* = 0.50 vs. 0.33), *p* = 0.055, though not with a high-intelligence expectancy (*M* = 0.42 vs. 0.32), *p* = 0.39. Overall, this pattern confirmed Hypothesis 4 in that in the presence of mostly expectancy-consistent information, high DFS were particularly attentive to the presence of expectancy-inconsistent behaviors.

However, when the majority of information did not match prior expectancies, high-DFS participants were more likely to recall expectancy-consistent information. When they expected Bob to be intelligent, but the behaviors mainly documented a lack of intelligence, high-DFS participants were likely to recall expectancy-consistent (i.e., intelligent) behaviors than either expectancy-inconsistent or neutral behaviors, *p* = 0.015 and *p* = 0.005 (**Figure 2**, center-left panel). Similarly, when expecting Bob to be of low intelligence, but the list of behaviors mainly showed the opposite, high-DFS participants were more likely to recall expectancy-consistent (i.e., unintelligent) behaviors than either expectancy-inconsistent or neutral behaviors, *p* = 0.027 and *p* = 0.001 (**Figure 2**, center-right panel). Pairwise comparisons between high-DFS and low-DFS participants showed that the former recalled more consistent behaviors when given a low-intelligence expectancy (*M* = 0.49 vs. 0.24), *p* = 0.007, though not with a high-intelligence expectancy (*M* = 0.46 vs. 0.44), *p* = 0.81. Confirming Hypothesis 5 and replicating Dijksterhuis et al. (1996), this pattern demonstrates that high desire-for-structure individuals are sometimes more attuned to expectancy-consistent than expectancy-inconsistent information, even when findings reported above showed that the pattern might be reversed under different circumstances. No other effects involving Behavior Type approached significance, nor did the same four-way interaction emerge when DFS was replaced with RLS.

#### Judgments

Using a 2 (Expectancy) × 2 (Majority of Behaviors) factorial analysis with either DFS or RLS as a continuous predictor showed that participants rated Bob as more intelligent when they expected him to be intelligent, both *F*(1,72) > 8.69, *p* < 0.005, $\eta_{p}^{2}$ > 0.10. Likewise, participants rated Bob as more intelligent when they received a list which included 12 intelligent behaviors instead of 12 unintelligent behaviors, both *F*(1,72) > 5.71, *p* < 0.02, $\eta_{p}^{2}$ > 0.07. DFS (but not RLS) interacted with Majority of Behaviors, *F*(1,72) = 4.34, *p* = 0.041, $\eta_{p}^{2}$ = 0.06. Regardless of participants’ expectancy (three-way *F* < 1), low-DFS participants (1 SD below mean) rated Bob to be more intelligent if the majority of the behaviors supported this conclusion compared to when the majority led to the opposite conclusion (*M* = 5.37 vs. 4.25), *p* = 0.002. However, among high-DFS participants (1 SD above mean), this comparison was not significant (*M* = 4.83 vs. 4.74), *p* = 0.78. No other effects materialized.

#### Relationship between Judgment and Recall

The ratio of expectancy-consistent behaviors to expectancy-inconsistent behaviors correlated somewhat positively with the intelligence rating when participants expected Bob to be intelligent and when the majority of the behaviors supported this conclusion, *r*(19) = 0.39, *p* = 0.095. However, the corresponding correlation was negative when participants expected Bob to be unintelligent with the majority of the behaviors supporting this conclusion, *r*(20) = –0.40, *p* = 0.092. That is, when expectancy and the majority of behaviors were aligned, relative recall of intelligent and unintelligent behavior was reflected in judgments. The strength of this relationship was weakened when expectancy and majority of evidence were not aligned, *r*(19) = 0.11, *p* = 0.66 and *r*(19) = -0.22, *p* = 0.36, respectively, though the direction of the coefficients remained unchanged^4^.

## Experiment 4

Though in Experiments 1–3 it was presumed that attentional processes are at the heart of the demonstrated memory and judgment effects, none of these studies provided any direct evidence. Thus, the goal of Experiment 4 was to replicate aspects of Experiment 3 and to tap the process of how individuals high or low in DFS allocate attention. Similar to Dijksterhuis et al. (1996), Experiment 4 measured how long participants wished to think about expectancy-consistent and expectancy-inconsistent behaviors, and which were either provided as part of a majority or a minority of behaviors. Note that previous research has established that expectancy-inconsistent information is particularly attention-grabbing (e.g., White and Carlston, 1983; Sherman et al., 1998). Specifically, Stern et al. (1984) demonstrated that individuals spend more time reading expectancy-inconsistent compared to expectancy-consistent behaviors, presumably to reconcile the behavior with their expectancy. However, consistent with Hypothesis 4, it was expected that high desire-for-structure individuals would attend primarily to expectancy-inconsistent information if the majority of the information provided was expectancy consistent. Conversely, and consistent with Hypothesis 5, high desire-for-structure individuals were expected to attend to expectancy-consistent information more than -inconsistent information when the majority of available information was expectancy-inconsistent.

### Participants

A total of 104 undergraduate students from a large introductory psychology course participated in this research in exchange for partial course credit. As in Experiment 1 participants were either from the bottom (*n* = 53) or top (*n* = 51) 20% of the distribution of the six-item short-version of the PNS scale (α = 0.86; Neuberg and Newsom, 1993), which all students completed at the beginning of the semester.

### Materials and Procedure

The procedure was similar to Experiment 3. Participants read a paragraph with a general description of Bob, followed by 24 behaviors, of which either 12 or 6 were consistent with the expectancy, and 6 or 12 were inconsistent with the expectancy, with 6 behaviors always being neutral. In contrast to Experiment 3, all participants read the same paragraph leading them to expect Bob to be highly intelligent. Participants were then presented with all 24 behaviors sequentially on a computer screen. Other than in the sequential presentation condition of Experiment 1, though, participants proceeded at their own pace such that they pressed a key to see one behavior after the other. The time between when the behavior appearing on screen and participants used a keystroke to advance to the next behavior was recorded by the computer and served as a measure of reading time.

Based on 12 intelligent and 12 unintelligent behaviors, again two high intelligence lists (12 intelligent and 6 unintelligent behaviors) and two low intelligence lists (12 unintelligent and 6 intelligent behaviors) were constructed. As noted before, this was done to ensure that, across participants, each behavior would be used equally often as part of the group of six behaviors in the minority. Each list was presented in the same random order, though the first behavior was always expectancy-consistent.

Following a distractor, participants engaged in the 3-min recall task and complete the same trait ratings as in Experiment 3. However, the order of these two tasks was counterbalanced, with half receiving the recall task first, and half receiving the judgment task first^5^.

### Results

#### Reading Times^6^

**FIGURE 3:**
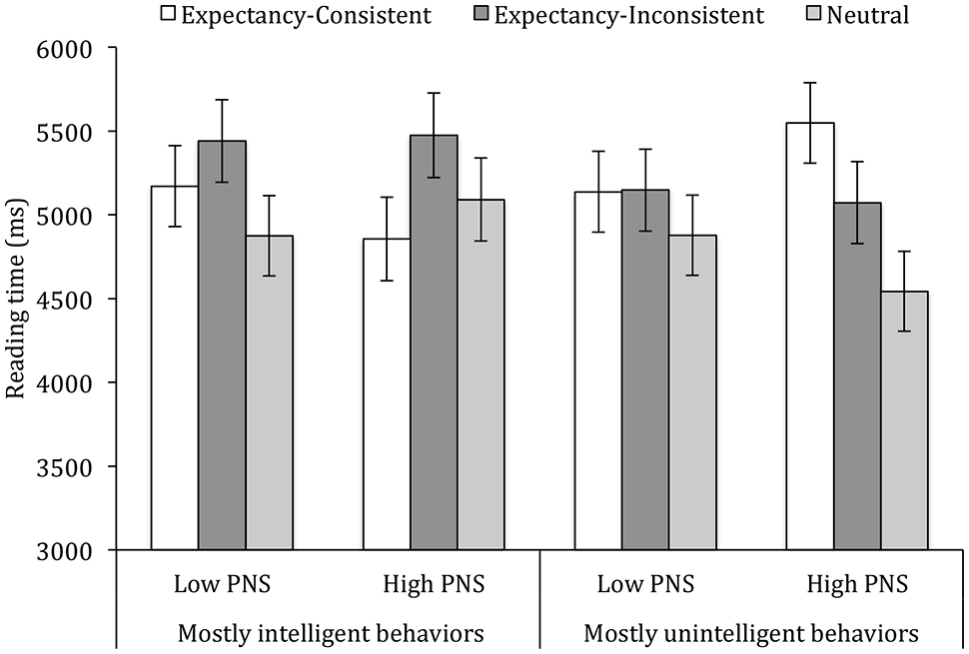
**Reading times for expectancy-consistent, expectancy-inconsistent and neutral behaviors as a function of majority of behaviors and PNS (Experiment 4).** Bars indicate one standard error above and below the mean.

A 2 (PNS) × 2 (Majority of Behaviors) × 2 (List version) × 3 (Behavior Type) analysis revealed that participants spent more time on expectancy-consistent (i.e., intelligent) behaviors (*M* = 5.18 s) and expectancy-inconsistent (i.e., unintelligent) behaviors (*M* = 5.28 s) than on neutral behaviors (*M* = 4.85 s), *F*(2,71) = 6.15, *p* = 0.003, $\eta_{p}^{2}$ = 0.14. This effect was qualified by Majority of Behaviors, *F*(2,73) = 5.98, *p* = 0.004, $\eta_{p}^{2}$ = 0.14. More importantly, the three-way interaction involving Behavior Type, Majority of Behavior and PNS was significant, too, *F*(2,73) = 4.27, *p* = 0.018, $\eta_{p}^{2}$ = 0.10. As shown in **Figure 3**, when the majority of behavior descriptions was consistent with the initial expectancy that Bob was intelligent, all participants spent more time on expectancy-inconsistent behaviors, though the difference between consistent and inconsistent behaviors was only reliable for high-PNS individuals, *p* = 0.008, but not low-PNS individuals, *p* = 0.23. Conversely, when the majority of behaviors presented to participants was inconsistent with the initial expectancy, high-PNS individuals spent more time reading the expectancy-consistent than the expectancy-inconsistent behaviors, *p* = 0.035, though again low-PNS individuals showed no difference, *p* = 0.96. This pattern confirmed that, depending on the informational context, individuals high in a DFS are variably more sensitive to expectancy-consistent or expectancy-inconsistent information. Note, though, that PNS seemed to mainly influence the relative time participants devoted to different kinds of items: in no case did reading times for low-PNS or high-PNS participants differ reliably, all *p* < 0.23.

#### Recall

Again, it was observed that expectancy-consistent and expectancy-inconsistent behaviors were more likely to be recalled than neutral behaviors, *F*(2,87) = 14.93, *p* < 0.001, $\eta_{p}^{2}$ = 0.26, which was qualified by Majority of Behaviors, *F*(2,87) = 7.30, *p* = 0.001, $\eta_{p}^{2}$ = 0.14. Critically, there was also a Behavior Type x Majority of Behaviors x PNS three-way interaction, *F*(2,87) = 2.90, *p* = 0.060, $\eta_{p}^{2}$ = 0.06. When the majority of behaviors supported participants’ expectancy, high-PNS individuals were more likely to recall expectancy-inconsistent behaviors rather than expectancy-consistent ones (*M* = 0.50 vs. 0.42), *p* = 0.040, whereas this was not true for low PNS participants (*M* = 0.45 vs. 0.50), *p* = 0.18. However, when the majority of behaviors was inconsistent with participants’ expectancy, high-PNS participants recalled expectancy-consistent behaviors better than expectancy-inconsistent ones (*M* = 0.48 vs. 0.33), *p* < 0.001, though this was also true for low-PNS individuals (*M* = 0.45 vs. 0.36), *p* = 0.017.

#### Judgment

Participants who had seen a majority of intelligent behaviors considered Bob more intelligent, *F*(1,88) = 6.73, *p* = 0.011, $\eta_{p}^{2}$ = 0.07. The effect was somewhat qualified by PNS, *F*(1,88) = 2.74, *p* = 0.10, $\eta_{p}^{2}$ = 0.03, though closer inspection revealed that low-PNS participants evaluated Bob as more intelligent when the majority of behaviors was intelligent compared to when it was not intelligent (*M* = 5.79 vs. 4.94), *p* = 0.003, though no such differences emerged for high-PNS participants (*M* = 5.26 vs. 5.05), *p* = 0.48. Low-PNS participants rated Bob as more intelligent than high-PNS participants when both prior expectancy and the majority of behaviors led to the same conclusion (*M* = 5.79 vs. 5.26), *p* = 0.055.

#### Correlations

When reading times for consistent behaviors were divided by reading times for inconsistent behaviors, the resulting ratio was positively correlated with the relatively greater recall of consistent to inconsistent behaviors, *r*(81) = 0.28, *p* = 0.014, replicating Stern et al. (1984). This is consistent with the idea that more effortful encoding is correlated with better recall, though this association was only significant when recall followed the judgment task, *r*(42) = 0.33, *p* = 0.032, not when these tasks were reversed, *r*(39) = 0.19, *p* = 0.25. The ratio for reading times was uncorrelated with intelligence judgments, *r*(81) = –0.05, *p* = 0.65. Similarly, the ratio of intelligent-to-unintelligent behaviors recalled was not related to intelligence judgments of, *r*(103) = -0.02, and this coefficient did not vary as a function of the order in which participants worked on the recall and judgment tasks^7^.

## Discussion

The present series of experiments sought to investigate if need for structure necessarily implies closed-mindedness, which was understood as individuals disregarding new and unexpected information. Much of the existing literature on need for closure and need for structure does suggest that individuals high in need for closure are more likely to rely on stereotypes (Neuberg and Newsom, 1993), are more likely to categorize others (Moskowitz, 1993), and in the process pay little attention to and not remember information that is not already congruent with their expectations (Dijksterhuis et al., 1996; see also Driscoll et al., 1991). Whereas categorical processing undoubtedly can have this effect, as confirmed by the last two experiments reported here, the present research shows that a heavy reliance on social categorization might also result, paradoxically, in an increased sensitivity to the new and unexpected. Participants strongly motivated to reduce ambiguity and increase structure through categorization were *more* likely to recall expectancy-inconsistent information (Experiments 1–4). This enhanced memory then prompted these participants to moderate their judgments, such that evaluations were more less stereotypical or less extreme than for participants low in DFS (Experiments 2–4). In other words, the present research confirmed that high DFS does not *necessarily* imply closed-mindedness.

This conclusion is supported by two important insights. First, earlier investigations by Dijksterhuis et al. (1996; as well as Driscoll et al., 1991) did not actually examine whether individuals high or low in need for structure varied in their inclination to engage in to categorical processing or not (but see Moskowitz, 1993). Investigators provided participants with explicit category information beforehand, which encouraged all participants to engage in categorical processing and, in the process, reduced if not eliminated any inter-individual variation in the inclination to do so. Thus, the Dijksterhuis et al. (1996) findings are clearly limited. When participants were given a task that was able to reveal inter-individual variability in the inclination to engage in categorical procession, high need for structure was related to better recall for impression-inconsistent information (Experiments 1 and 2), and more moderate judgments (Experiment 2).

Second, by replicating the findings by Dijksterhuis et al. (1996) the present research confirmed that DFS *can* produce closed-mindedness, precisely when individuals engage in categorical processing. Yet, the present research demonstrates that the consequences of categorical processing and category-based expectancies are much more context dependent than previously envisioned by research on need for structure. Extending previous theorizing (Johnston and Hawley, 1994; Sherman et al., 1998) the present studies demonstrated that expectancies direct attention in a way that is most likely to facilitate information processing given the affordances of the situation. It was argued that for individuals high in desire-for-structure, categorical processing should direct attention to that which cannot be taken for granted in a given situation. When even a cursory inspection had to reveal that the majority of available information did not match the expectancy, participants focused more on expectancy-consistent information, and recalled it better. This pattern is consistent with the notion that encoding of expected information is facilitated by a well-developed conceptual structure that allows perceivers to link such information to what is already known about a target (e.g., Macrae and Bodenhausen, 2000; Skowronski et al., 2013). However, broader conclusion is that focusing on expectancy-consistent information allows high desire-for-structure individuals to navigate a stream of ambiguous information, most of which challenges their *a priori* expectancy. Under these circumstances, a focus on what is already known is much more likely to reduce subjective feelings of uncertainty and increase cognitive structure.

However, when the majority of the encountered evidence is supportive of one’s expectancy, uncertainty is not reduced and cognitive structure is not enhanced when participants dwell on expectancy-consistent information (cf. Sherman et al., 1998, 2004). Rather, in order to increase structure, participants high in DFS are more likely to attend to information that does challenge their prior expectancy, possibly because thinking about such information might allow it to be reconciled with the expectancy. For instance, if high desire-for-structure participants expect Bob to be highly intelligent, but learn that he is also a high school dropout, they might consider that he was bored and under-challenged, and that he was wasting his time in public school. Thus, attending to expectancy-inconsistent information might be driven as much by a motivation to reduce ambiguity and increase cognitive structure as is attending to expectancy-consistent information.

At a more general level, the present research suggests that the consequences of a high need for structure for information processing must always be theorized within the specific context of the task affordances. Much research has reported what are ultimately main effects for need for structure/need for closure (e.g., Neuberg and Newsom, 1993; Kruglanski and Webster, 1996; Kruglanski, 2004). However, the present Experiments 3 and 4 demonstrated that variations in the distribution of the available information alone can dramatically alter the consequences of individual differences in need for structure. There is a long tradition in psychology which argues that thinking about the social world cannot be examined in isolation from the characteristics of the social world itself (see Fiedler, 2007 for a recent review). Likewise, it is argued here that the consequences of need for structure for especially information allocation cannot be determined confidently without knowing individuals’ information ecology, i.e., the nature and distribution of information in their social environment.

Once one acknowledges the critical role of information ecology, it is evident that Dijksterhuis et al. (1996) tackled a very specific situation, namely, one in which participants were given one third expectancy-consistency, one third expectancy-inconsistent and one third neutral information (see also Driscoll et al., 1991). Surely, this specific distribution of information was employed to avoid possible confounds resulting from the differential distribution of expectancy-consistent and expectancy-inconsistent (Hastie and Kumar, 1979; Hemsley and Marmurek, 1982; Bargh and Thein, 1985). However, like more recent studies examining the impact of need for closure on category-relevant information (e.g., Kossowska et al., 2012a,b), this “thirds”-approach severely limits what might be learned about the implications of need for structure for attention allocation specifically, and information processing in general. Hence, researchers are encouraged to take information ecology into consideration when examining need for structure effects.

Critically, the present experiments show that the increased attentiveness and sensitivity to expectancy-consistent information were mainly a function of only one of two dimensions of the PNS construct, the desire to structure one’s life and one’s thinking about one’s social environment in a simple way (Neuberg and Newsom, 1993). Though the recruitment of participants for Experiments 1 and 4 did occur based on a combined PNS score, Experiments 1–3 showed that whenever DFS and RLS were assessed within the same experimental session, there was evidence for focal effects to emerge for DFS, but not necessarily for RLS. This supports the present contention that it is participants’ motivation to engage in social categorization as a primary cognition-simplifying process that is responsible for the observed effects, rather than a typically aversive RLS. As such, the present research adds to the literature relating specific facets of need for structure or need for closure to specific social-cognitive phenomena (e.g., Roets and Van Hiel, 2007; Cavazos et al., 2012).

Though the present research contributes to a more complete understanding on the implications of need for structure for the processing of expectancy-inconsistent information, it also poses a number of questions. Prominently, the nature of the process as to why expectancy-inconsistent information is more memorable than expectancy-consistent information is not entirely transparent. It is typically assumed that people are motivated to reconcile incongruent observations with existing knowledge, resulting in a memory advantage for this type of information (e.g., Srull et al., 1985; Stangor and McMillan, 1992). Yet, evidence tapping this process directly remains elusive (Heider et al., 2007; Skowronski et al., 2013). Therefore, it is not entirely clear as to why exactly high need-for-structure individuals did exhibit better recall memory for expectancy-inconsistent information. This question is particularly pertinent because in Experiment 1 such individuals did not exhibit a recognition advantage. Moreover, in the same study high DFS participants revealed lower recall memory for expectancy-consistent information. Even though this finding did not replicate in Experiments 2–4, the issue clearly deserves further study. That high need-for-closure participants in Experiment 1 did rely on categorical processing was evident in the elevated false alarms rates for newly presented expectancy-consistent information—a telltale sign that participants were guided by category-based expectancies. Somewhat confusingly, though, there was some evidence that the same participants also falsely recognized expectancy-inconsistent information as evidenced in the response bias scores obtained as part of Experiment 1. This shows that participants were especially attuned to expectancy-*relevant* information, regardless of whether consistent or inconsistent with their expectancies; however, the pattern highlights that the underlying process is not yet clear (cf. Skowronski et al., 2013).

Experiments 2 and 3, though not Experiment 4, demonstrated that in the present paradigm recall memory and judgments about the target were related. To the extent that participants were more likely to recall evidence that contradicted the overall impression, they were more moderate in their evaluation of the target. That is, just as Dijksterhuis et al. (1996) demonstrated that high need-for-structure participants’ lower recall of stereotype-inconsistent information led to more stereotypical judgments, the previous research demonstrated that the same recall-based judgment process might lead to the opposite tendency.

Though it should not be surprising that participants relied on their memory for specific behaviors when evaluating a target person whom they had only learned about minutes beforehand, arguably demonstrating a link between encoding processes and recall is somewhat more impressive. Experiment 4 showed that the longer participants devoted to thinking about expectancy-inconsistent relative to expectancy-consistent information, the more likely were they recall the former relative to the latter, conceptually replicating Stern et al. (1984). Yet, there was little evidence of a link between reading times and judgment. Although this mirrors Dijksterhuis et al. (1996), it stands to reason that the self-paced exposure to expectancy-consistent or expectancy-inconsistent information may have disrupted any link between recall memory and memory-based judgment, possibly because participants formed judgments on-line (Hastie and Park, 1986).

The present research faces at least two important limitations. First, Kossowska et al. (2012b) recently determined that the effects of need for structure vary by age. Specifically, among younger adults (20–26 years) higher levels of need for structure were linked to greater categorical processing such that high need-for-structure participants were more likely to recall expectancy-consistent information than low need-for-structure participants, whereas this relationship was reversed among older adults (65 years and older). Moreover, these authors observed that among older adults, high levels of need for structure were linked to better memory for expectancy-irrelevant (neutral) information (see also Kossowska et al., 2012a). This aging-related dynamic was not captured in the present series of studies, where the focus was exclusively on a college-aged population^8^. Note, that these authors relied on a task that resembled Driscoll et al. (1991) and Dijksterhuis et al. (1996) in that it provided participants with a category label about whom participants subsequently learned equal numbers of expectancy-consistent, expectancy-inconsistent and neutral characteristics (see above).

A second limitation arises from the fact that the present research did not measure the participants’ subjective ability to fulfill their need for closure. Research by Bar-Tal et al. (1997) and Kossowska and Bar-Tal (2013) have documented that sometimes individuals high in need for structure feel unable to employ a processing style that will reduce uncertainty and ambiguity. Using the same paradigm as Kossowska et al. (2012b), Kossowska and Bar-Tal (2013, Study 1) demonstrated that only among participants high in “ability to achieve closure” did need for structure predict lower recall of expectancy-inconsistent information, whereas no such relationship existed for participants low on this ability dimension. In other words, ability to achieve closure likely serves as a moderator, which qualifies the strength, but not the direction, of any effects of need for structure on memory for expectancy-consistent and expectancy-inconsistent information^9^. This implies that the omission of this variable from present investigation does not call into question any of its conclusions, even when it is clear that an additional level of complexity exists—a notion further corroborated by the observation that need for structure and ability to achieve closure are uncorrelated (Kossowska and Bar-Tal, 2013).

Still, future research should examine to what extent ability to achieve closure qualifies any need for structure effects in a paradigm in which, unlike in Kossowska and Bar-Tal (2013, Study 1) and Dijksterhuis et al. (1996), participants are not provided with an *a priori* category label by the experimenter. Likewise, it would be interesting to see if ability to achieve closure does qualify need for structure effects when the information ecology is varied. Employing this variable would also be of great theoretical interest because the effects demonstrated here have to be attributed to one of two dimensions of the PNS construct, namely DFS (Neuberg and Newsom, 1993). It would be important to know if effects of subjective ability are specific to this one dimension of whether it generalizes to all dimensions, especially if one considers other measure of need for closure, namely the need for closure scale (Webster and Kruglanski, 1994), distinguish a total of five sub-dimensions.

In closing, it is often thought that high need for structure ushers in categorical processing. When relying on categorical processing, a perceiver is always at risk of pigeonholing new acquaintances, of rendering them exchangeable members of a social category that has been activated in the perceiver’s mind. However, categorical processing may also provide perceivers with expectancies about newly encountered others in the first place. That is, perceivers who categorize and as a consequence hold expectancies must determine whether a particular piece of information about the new acquaintance is expected or unexpected. To the extent that a new acquaintance is not a typical member of an activated social category, high need-for-structure individuals may discover much unexpected information, which will grab their attention and, presumably, requires them to think. Ironically, this may commit high need-for-structure perceivers to investing considerable cognitive effort—very much contrary to their general inclination to reduce processing effort (Neuberg and Newsom, 1993; Kruglanski, 2004). Equally ironically, under such circumstances high need-for-cognition perceivers are more likely to incorporate information into their judgment, which contradicts an earlier expectancy about a target. That is, when high need-for-structure individuals enter a situation without clear expectancies or with an expectancy that is clearly not supported by the evidence, their tendency to over-rely on social categories may have at its roots the undoing of this very over-reliance. Information that does not fit will “stick out.”

## Conflict of Interest Statement

The author declares that the research was conducted in the absence of any commercial or financial relationships that could be construed as a potential conflict of interest.

## Footnotes

1 Pre-screening did not occur based on DFS only. However, because three of four DFS items were included in the six-item PNS measure and DFS and RLS are highly correlated (*r* > 0.54–0.74; Neuberg and Newsom, 1993), this recruitment procedure ensured that participants were very high or low in DFS.

2 Two outliers were removed from the recognition analyses because standardized residuals were smaller than -4.

3 When the two Majority of Behavior conditions were combined, the correlation between recall ratio and judgment was confirmed, *r*(96) = 0.24, *p* = 0.017.

4 When collapsing across expectancy conditions, when expectancy and majority of behaviors were aligned, the correlation between recall ratio and judgment was substantial, *r*(38) = 0.31, *p* = 0.06, but was significantly lower when expectancy and majority of behaviors were not aligned with each other, *r*(39) = 0.12, *p* = 0.47.

5 Participants also completed the PNS scale, and indicated their age and gender, but regrettably these data were lost.

6 Because of user error and technical complications, reading-time data for 22 participants were not recorded.

7 Reminiscent of Experiment 3, when participants’ expectancy and the majority of behaviors were aligned (i.e., participants expected Bob to be intelligent and saw a majority of intelligent behaviors), the correlation between recall and judgment was positive, *r*(54) = 0.17, *p* = 0.22, though not significant. However, when majority of behaviors and expectancy were not aligned, this was not the case, *r*(49) = -0.06, *p* = 0.68.

8 Kossowska et al. (2012b) reported that NFC was associated with lower recall for expectancy-inconsistent information, Study 1, *r* = -0.19 and Study 2, *r* = -0.06, though these coefficients were not significant. Separate correlations for younger and older adults were not provided.

9 Kossowska and Bar-Tal (2013) found that need for structure qualified the recall of neutral information, such that need for structure was related to greater recall when “ability to achieve closure” was low, but lower recall when “ability to achieve closure” was high.
